# High expression of circulating exosomal PD-L1 contributes to immune escape of hepatocellular carcinoma and immune clearance of chronic hepatitis B

**DOI:** 10.18632/aging.206020

**Published:** 2024-07-17

**Authors:** Xiaoqing Lin, Hui Shao, Yongzhi Tang, Qiupeng Wang, Zhenyu Yang, Hongwei Wu, Tongjing Xing

**Affiliations:** 1 Wenzhou Central Hospital, Dingli Clinical College of Wenzhou Medical University, Wenzhou Sixth People’s Hospital, Wenzhou, Zhejiang, China; 2 Taizhou Hospital of Zhejiang Province Affiliated to Wenzhou Medical University, Linhai, Zhejiang, China

**Keywords:** programmed death ligand-1, exosomes, chronic hepatitis B, hepatocellular carcinoma

## Abstract

Objective: To investigate the expression of programmed death ligand-1 (PD-L1) in circulating exosomes, and to define the role of exosomal PD-L1 in promoting immune escape mechanism during chronic hepatitis B infection (CHB) and related liver diseases.

Methods: The levels of PD-L1 expressed in exosomes were detected by ELISA. CD8+T cells were sorted and cytotoxicity test was assessed by flow cytometry. PD-L1 protein expression in hepatocellular carcinoma (HCC) and normal adjacent tissues were detected by immunohistochemistry.

Results: Circulating exosomal PD-L1 levels were significantly higher in patients with CHB and HCC than in healthy controls (F =7.46, P=0.001). Levels of CD107a on CD8+T cells in patients with CHB receiving PD-L1 blocking antibody were significantly lower than in patients receiving isotype blocking antibody (t = 4.96, P < 0.01). Levels of TNF-α in cell culture supernatants of the PD-L1 blocking antibody group were significantly higher than in the isotype blocking antibody group (t =5.92, P < 0.01). Compared with patients receiving isotype blocking antibody, levels of CD107a on CD8+T cells significantly increased in patients with HCC receiving anti-PD-L1 antibody (t = 3.51, P<0.05). Compared with adjacent tissues, the levels of PD-L1 protein expression in HCC tissues were slightly higher; however, no significant difference between the two groups was observed.

Conclusions: PD-L1 blockade in exosomes might promote the cytotoxic function of CD8+T cells and inhibit immune evasion during progression of HCC. Blocking PD-L1 in exosomes reduced the cytotoxic function of CD8+T cells in patients with CHB while enhancing the production of proinflammatory cytokines.

## INTRODUCTION

Hepatocellular carcinoma (HCC) is the second-leading cause of cancer-related mortalities worldwide. About 745,000 HCC patients die each year, with patients in China accounting for more than 50% of affected individuals [1]. Chronic hepatitis B (HBV) and hepatitis C (HCV) infection are the primary causes of HCC. In China, a majority of HCC cases are primarily due to chronic HBV infection. Chronically infected HBV patients suffer from repeated episodes of intrahepatic inflammation, leading to the onset of liver cirrhosis and HCC [2]. Recent studies have reported that the immunosuppressive tumor microenvironment induced by liver cancer cells promotes immune evasion and contributes to the development of HCC [3, 4]. Major advances have been made in the identification of novel molecular and immune targeted therapies for HCC. However, our knowledge of the effectiveness of these therapies in patients with advanced liver cancer is limited [5]. Therefore, elucidating the immune pathogenesis of HCC is vital for making advancements in new therapies and prognosis evaluation methods.

Exosomes are double-layer lipid inclusion structures with a diameter of 30-200 nm that are present in body fluids and can be secreted by a variety of cell types in the human body [6]. Exosomes can regulate the biological activity of recipient cells through the secretion of proteins, nucleic acids, and lipids carried by them [6]. A large number of studies have shown that exosomes play an important role in driving tumor progression and metastasis, including during HCC [7, 8]. Rao et al. found that tumor exosomes carrying a hepatocyte antigen could induce robust dendritic cell responses and inhibit tumor growth and metastasis [9]. Zhang et al., meanwhile, reported that exosomal transfer of transforming growth factor β (TGF-β) derived from hepatoma cells induced tumor infiltrating NK cell dysfunction via the TGF-β/SMAD pathway, leading to immune escape [10]. During chronic HBV infection, exosomes can directly participate in HBV replication and regulate the host immune response to infection [11]. Ultimately, secretion of exosomes has the ability to modulate multiple facets of chronic HBV infection and HCC. Specifically, delivery of various exosomal products can have immune stimulatory or suppressive effects and can contribute to immune evasion or persistence. Thus, it is vital to further our understanding of the immunomodulatory effects of exosomes in contributing to HCC pathogenesis to aid in the identification of novel therapeutics for HCC.

The programmed cell death protein 1 (PD-1) signaling pathway, composed of PD-1 and its ligands programmed cell death ligand 1 (PD-L1) and programmed cell death ligand 2 (PD-L2), play important roles in maintaining peripheral immune tolerance [12]. This is especially true in cancer and chronic infection, where engagement of the PD-1/PD-L1/2 pathway inhibits T cell–mediated immune responses against tumors and pathogens. PD-L1 is expressed not only in cancer cells but also in exosomes derived from cancer cells [13]. Exosome-derived PD-L1 can directly bind to T cells, inhibit their function, and promote tumor growth and immune escape [14]. However, the expression of PD-L1 in the exosomes of patients with chronic HBV and related HCC and its role in mediating immune escape have remained largely unstudied. Herein, we investigated the expression level of PD-L1 in circulating exosomes in patients with chronic HBV and related HCC. The effects of circulating exosomes on CD8^+^T cell functions and responses were investigated to identify the role exosomal PD-L1 plays in the pathogenesis of HBV and HCC.

## MATERIALS AND METHODS

### Sample collection and participant inclusion criteria

Peripheral blood was collected with serum separator tube or sodium citrate tube from patients with chronic hepatitis B (CHB), and HCC who were hospitalized at Taizhou Hospital, affiliated with Wenzhou Medical University, between January 2018 and November 2021. A total of 47 patients and 23 healthy controls were included in our study. The average age of the patients was 53 (27–82) years of age. Among them, 24 patients had HBV-related HCC, 23 patients had CHB. Table 1 shows the baseline data of patients. Diagnoses and disease staging were in accordance with the guidelines for the prevention and treatment of chronic HBV (2015 version) [15], and the guidelines for the diagnosis and treatment of primary liver cancer (2019 version) [16]. Patients with HCC due to HCV, HIV, alcohol abuse, and autoimmunity, or patients with cholangiocarcinoma and metastatic liver tumors were excluded from the study. Paired paraffin-embedded liver cancer and adjacent healthy tissues were collected from patients with chronic HBV-related HCC. All patients were treatment naïve and had not undergone radiotherapy or chemotherapy prior to sample collection.

**Table 1:** Baseline data of patients and healthy controls.

	**CHB (23)**	**HCC (24)**	**HC (23)**
Sex (m/f)	18/5	22/2	10/13
Age (yr)	47 ± 12	57 ± 11	41± 12
ALT (U/L)	398.3 ± 402.9	92.3 ± 223.5	19.9 ±8.9
HBV DNA (Log10IU/mL)	6.40 ± 1.62	2.95 ± 2.03	-
TBil (umol/L)	43.7±62.1	35.9±36.8	13.4±2.8
ALB (g/L)	35.5±6.1	36.8±7.0	47.2± 2.4

CHB, chronic hepatitis B; HCC, Hepatocellular carcinoma; HC, healthy controls.

### Isolation and identification of exosomes

The serum was separated and frozen at 1 ml in each tube at – 80° C. The circulating exosomes were isolated using the Exo Quick™ exosome separation Kit (SBI Comp., CA, USA) according to the manufacturer’s instructions. Briefly, frozen samples were thawed and mixed. Samples were centrifuged at 4° C, 13000 rpm for 15 min, and supernatants were transferred to a new tube. Exosome precision solution was added to the samples, followed by a 30 min incubation at 4° C. After incubation, samples were centrifugated, supernatants discarded, and collected precipitations were resuspended with 100 μl PBS and stored at −80° C until the time of analysis. The morphology and particle size distribution of isolated exosomes were identified by NanoSight particle size density distribution analysis and transmission electron microscopy. Nanoparticle Tracking Analysis (NTA) analysis was performed using ZetaView PMX 110 (Particle Metrix, Meerbusch, Germany). Polystyrene microspheres (110 nm) were used for calibration, samples were diluted with 1X PBS buffer (Biological Industries, Kibbutz Beit-Haemek, Israel) (dilution ratio 1:1000) and for detection. 10 μl sample were dropped on the formvar carton-coated copper grids. After staining with 10 μl uranyl acetate and dried, grids were visualized using FEI Quanta 250 Transmission electron microscope (TEM). A human PD-L1 enzyme-linked immunosorbent assay (ELISA) kit (Thermo Fisher Scientific, MA, USA) was used to detect the expression level of PD-L1 in exosomes, performed according to the manufacturer’s instructions.

### Western blot

Exosome suspension was lysated and separated by SDS-PAGE gel. Proteins were transferred to PVDF membranes. Membranes were blocked in TBST solution containing 10% skim milk. Antibodies specific for CD63 (1:1000) (Abcam, Cambridge, UK) and TSG101 (1:1000) (Abcam, Cambridge, UK) were added and incubated overnight at 4° C. Membranes were washed with PBST solution, followed by probing with HRP-conjugated secondary antibodies (Arigo, Taiwan, China) at room temperature for 2 h. The Immobilon Western HRP Luminescent reagent (Millipore Corp., MA, USA) was used for color development. A Canon LiDE120 scanner was used for image scanning.

### Sorting of peripheral CD8^+^T cells

Peripheral blood mononuclear cells (PBMCs) were routinely isolated using Ficoll-Hypaque methods. Briefly, isolated PBMCs were washed with PBS and centrifuged at 4° C, 1000 rpm for 5 min. Next, cells were resuspended with 100 μl PBS and stained with FITC mouse anti-human CD3 (BioLegend, CA, USA) and PE mouse anti-human CD8 (BD Pharmingen, CA, USA) antibodies for 20 min in the dark. Samples were centrifuged at 4° C, 1000 rpm for 5 min, and the supernatant was discarded. Lastly, cells were washed and resuspended with PBS. CD8^+^T cells were sorted with a FACSAria III flow cytometer (BD Pharmingen, CA, USA).

### Functional analysis of peripheral CD8^+^T cells

PD-L1 blocking antibody (5 μg/mL) or isotype control antibody (5 μg/mL) (Bio X Cell, NH, USA) were cultured with exosomes isolated from the peripheral blood and incubated for 24 hours. Separately, CD8^+^T cells (5 × 10^4^ cells) were plated in 96-well plates and stimulated with anti-CD3 (2 μg/mL) and anti-CD28 antibodies (2 μg/mL) (BD Pharmingen, NJ, USA) for 24 hours. CD8^+^T cells were separated into one of two treatment groups. One group was cultured with exosomes treated with anti-PD-L1 antibody, and the other group was cultured with exosomes treated with isotype control antibody. After 48 hours, cells and culture supernatants were collected and stored at −80° C for downstream analyses. Cytokines in culture supernatants were detected by enzyme-linked immunosorbent assay (ELISA).

### Detection of CD107a in CD8^+^T cells by flow cytometry

The cytotoxic function of CD8^+^T cells was tested via a cytotoxicity test kit (BD Pharmingen, NJ, USA) to measure CD107a expression and was performed according to the manufacturer’s instructions. After fixation and permeabilization, cells were resuspended with PBS and stained with CD107a antibody (BD Pharmingen, NJ, USA) in the dark for 20 min. Cells were washed and resuspended with PBS, and CD107a expression levels were detected by CytoFLEX flow cytometry (Beckman Coulter, CA, USA).

### Detection of PD-L1 by immunohistochemistry

Immunohistochemistry was performed using the conventional streptavidin-peroxidase method. Paraffin sections of HCC and adjacent tissues were dehydrated, blocked, and incubated with anti-PD-L1 antibody (1:400; Abways Technology, Inc., Shanghai, China) overnight. The following day, slides were washed with PBS and incubated with secondary antibody for 2.5 hours. After washing with PBS, slides were incubated in color developing solution for 15 min. Next, slides were washed with a chromogenic solution and incubated in hematoxylin dye for 40 seconds. After a final washing, slides were de-stained using a 1% HCl solution for 1 second. Positive PD-L1 staining was observed under a DM 500 microscope (Leica, Wetzlar, Germany).

### Statistical analysis

Data analysis was performed using SPSS 17.0 statistical software (SPSS, Inc., IL, USA). Student’s *t*-tests were performed to compare two groups. The analysis of variance and the SNK-q test were used to compare multiple groups. A X^2^ test was used to analyze counting data. Pearson’s correlation analysis was used to perform correlation analysis of the data. P < 0.05 was considered to be statistically significant for all of the analyses performed.

## RESULTS

### Isolation and identification of circulating exosomes from patients with HBV-related liver disease

**Figure 1:**
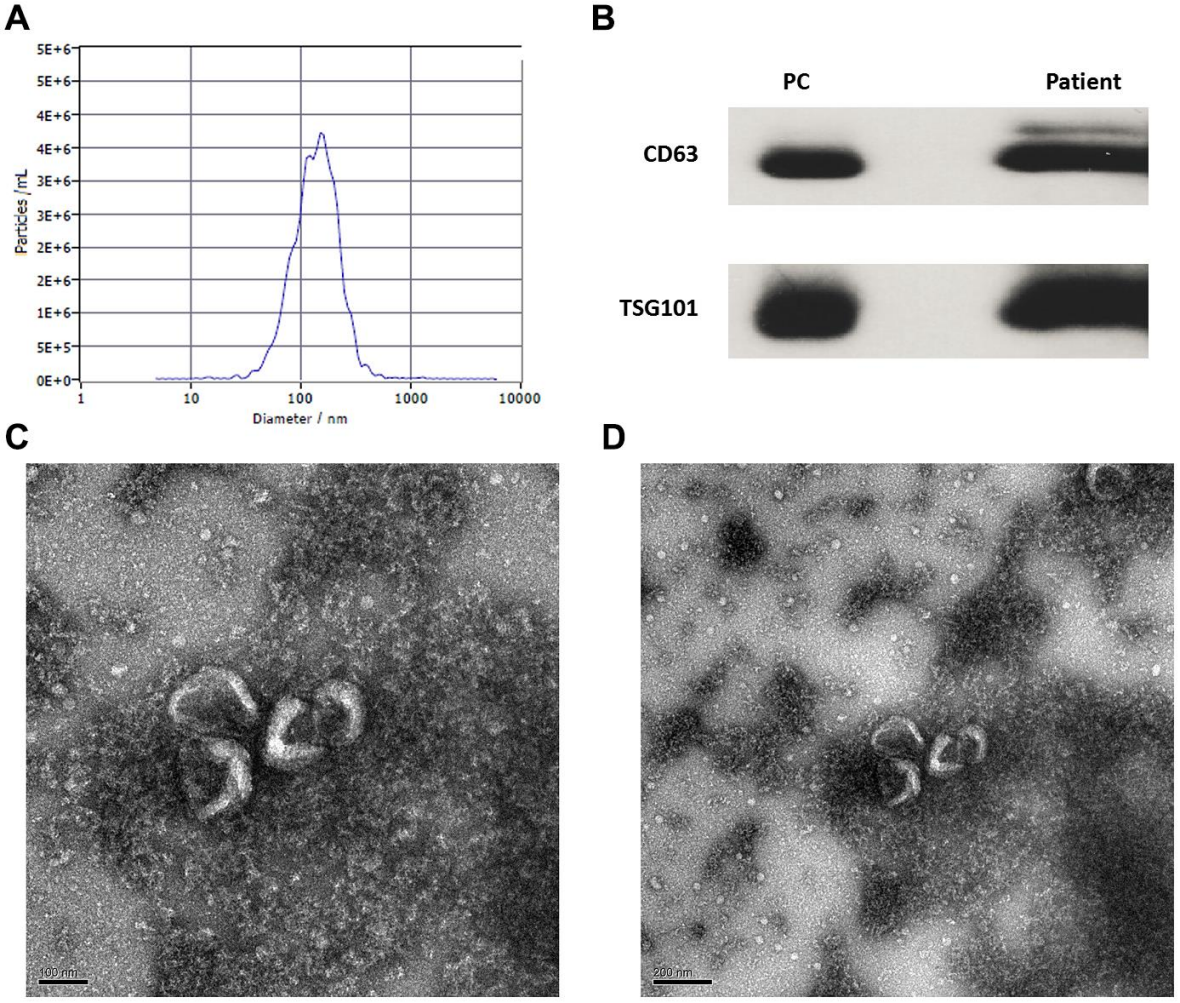
**Identification and isolation of circulating exosomes of patients with CHB and hepatocellular carcinoma.** (**A**) The particle size and concentration distribution of exosomes; (**B**) The wb images of CD63 and TSG101 markers (PC: positive control); (**C**) The exosomes observed by transmission electron microscope (100 nm); (**D**) The exosomes observed by transmission electron microscope (200 nm).

Circulating exosomes from patients with HBV-related liver diseases were isolated and identified by Western blot (wb), NanoSight particle size density distribution analysis, and transmission electron microscopy. The particle size of isolated exosomes was 134.5 ± 6.4 nm, and was consistent with previous reports [17] (Figure 1A). The wb images of CD63 and TSG101 were showed as Figure 1B. Exosomes isolated were round or oval with a complete membrane structure as determined by transmission electron microscopy (Figure 1C, 1D).

### Expression of PD-L1 in circulating exosomes of patients with HBV-related liver diseases

**Figure 2:**
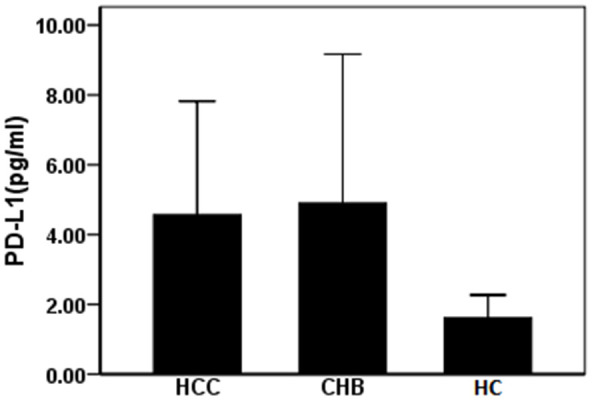
**Expression of PD-L1 in serum exosomes of patients with HBV related liver diseases.** CHB: chronic hepatitis B; HCC: hepatocellular carcinoma; HC: healthy controls.

A total of 47 patients and 23 healthy controls (HC) were included in our study. Among them, 24 patients had HBV-related HCC, 23 patients had CHB. The average age of the HCC and CHB patients was 58 (37–82) and 47 (27–82) years of age. Table 1 shows the baseline data of patients and healthy controls. The expression level of PD-L1 in circulating exosomes was detected by ELISA. We detected 4.90±4.26, 4.57±3.25 pg/mL, and 1.61±0.66 pg/mL of PD-L1 in exosomes in patients with CHB, HCC, and HC, respectively. Compared with HC, levels of PD-L1 in circulating exosomes in patients with CHB and HCC were significantly increased (*F* =7.46, *P=*0.001). However, expression levels of PD-L1 in circulating exosomes between patients with CHB and HCC were not statistically significant (Figure 2). These data suggest that circulating exosomal PD-L1 expression increases in patients with CHB and HCC.

### Evaluating the effect of exosomal PD-L1 on CD8^+^T cell functionality in patients with HCC

**Figure 3:**
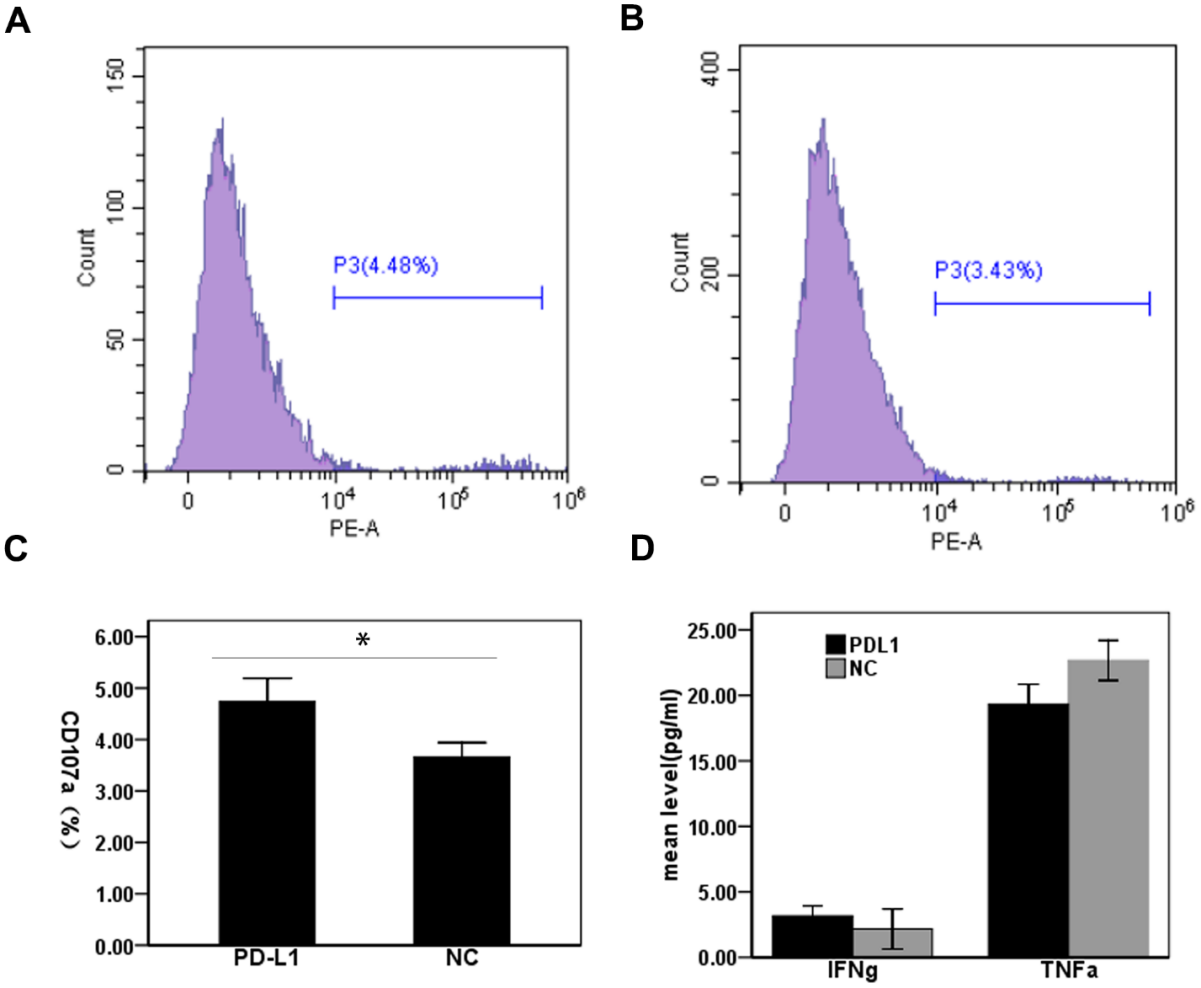
**Effect of PD-L1 in circulating exosomes on CD8+T cell cytotoxicity in patients with hepatocellular carcinoma.** (**A**) PD-L1 antibody blocking; (**B**) Isotype control antibody blocking; (**C**) Comparison of CD107a expression levels between two groups; (**D**) Comparison of cytokine expression levels between two groups.

To determine the effect of exosomal PD-L1 expression on CD8^+^T cell responses during HCC, we measured CD107a expression in CD8^+^T cells from HCC patients by flow cytometry. Two groups were cultured with exosomes treated with anti-PD-L1 antibody and with isotype control antibody. Expression levels of CD107a in CD8^+^T cells treated with anti-PD-L1 antibody blockade were significantly higher than that in T cells stimulated in the presence of the isotype control antibody-blocking antibody (4.74 ± 0.45% vs. 3.66 ± 0.28%) (t = 3.51, P <0.05) (Figure 3A–3C). Next, we assessed proinflammatory cytokine production in cell culture supernatants from our T cell cultures by ELISA. Levels of IFN-γ in cell culture supernatants from anti-PD-L1-treated cultures were slightly increased compared to isotype control treated cultures (3.17 ± 0.76 vs. 2.17 ± 1.52 pg/mL), with no significant differences observed between treatment groups (t = 1.01, P > 0.05) (Figure 3D). Interestingly, levels of TNF-α in anti-PD-L1-treated cultures were slightly decreased compared to isotype control treated cultures (19.33 ± 1.52 vs. 22.67 ± 1.53 pg/mL), with no significant differences observed between treatment groups (t = 2.67, P > 0.05) (Figure 3D). Cumulatively, these data demonstrate that exosomal PD-L1 does alter CD8^+^T cell cytotoxic function but not the cytokine secretory function of CD8^+^T cell in HCC patients.

### Evaluating the effect of exosomal PD-L1 expression on CD8^+^T cell function in patients with CHB

**Figure 4:**
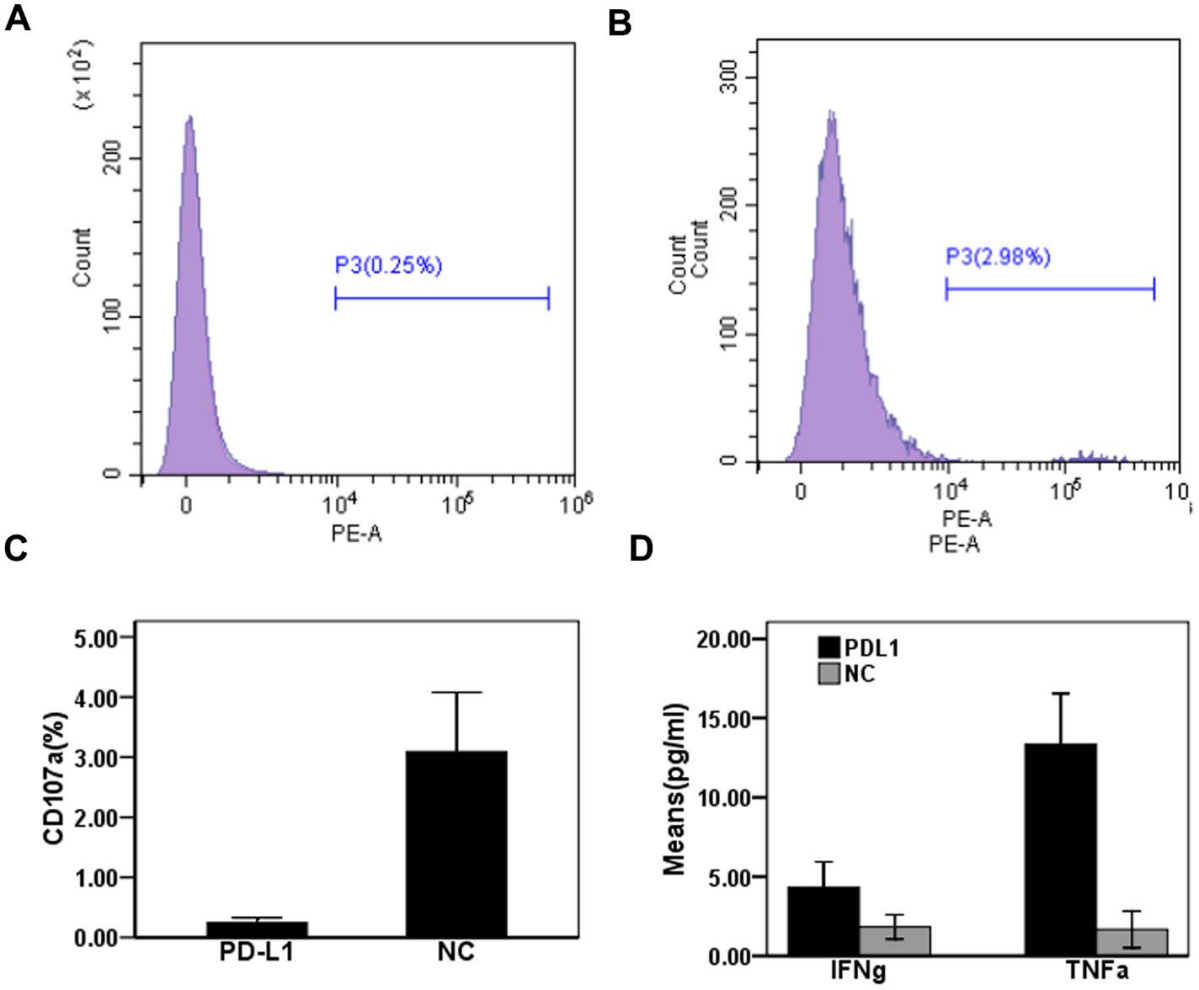
**Effect of PD-L1 in circulating exosomes on CD8+T cytotoxic function in patients with chronic hepatitis B.** (**A**) PD-L1 antibody blocking; (**B**) Isotype control antibody blocking; (**C**) Comparison of CD107a expression levels between two groups; (**D**) Comparison of cytokine expression levels between two groups.

To determine the effect of exosomal PD-L1 expression on CD8^+^T cell responses during chronic HBV infection, we measured CD107a expression in CD8^+^T cells from patients with CHB using flow cytometry. Expression levels of CD107a on CD8^+^T cells in the anti-PD-L1 antibody blocking group were significantly lower than those in the isotype control antibody blocking group (0.25 ± 0.08% vs. 3.09 ± 0.99%) (t = 4.96, P < 0.01) (Figure 4A–4C). The production of cytokines in cell culture supernatants were detected by ELISA. Levels of IFN-γ in cell culture supernatants from anti-PD-L1-treated cultures were slightly increased compared to isotype control treated cultures (4.33 ± 1.61 pg/mL vs. 1.83 ± 0.76 pg/mL) (t = 2.43, P = 0.07) (Figure 4D), while levels of TNF-α in anti-PD-L1 treated cultures were significantly increased compared to isotype control treated cultures (13.33 ± 3.21 vs. 1.67 ± 1.15 pg/mL) (t = 5.92, P = 0.004) (Figure 4D). These results suggest that exosomal PD-L1 alters both the cytotoxic and secretory functions of CD8^+^T cell in patients with CHB.

### Assessing the expression of PD-L1 in cancer and adjacent tissues of HCC patients

**Figure 5:**
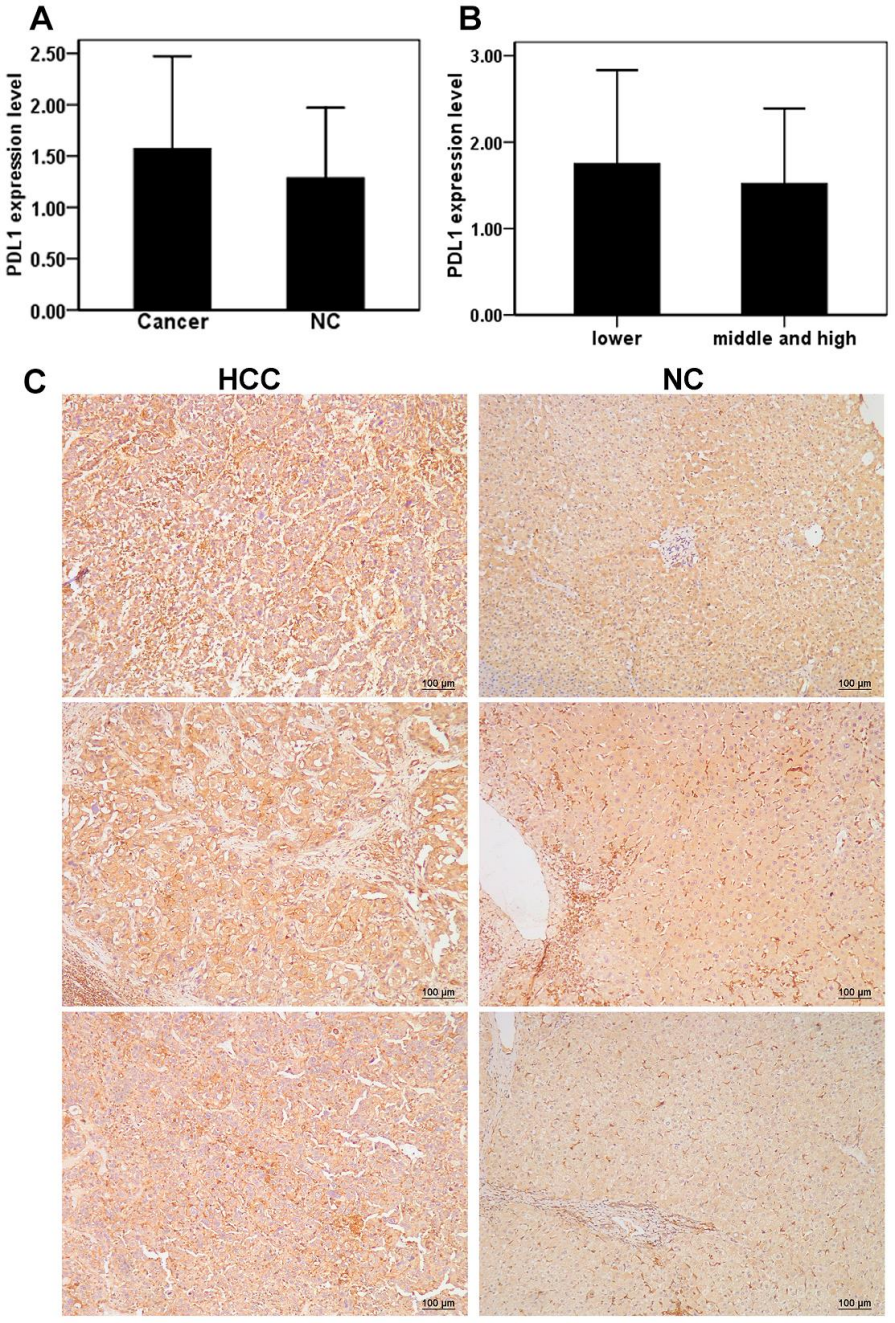
**Expression of PD-L1 in cancer tissues and adjacent tissues of HCC patients.** (**A**) The level of PD-L1 in hepatocellular carcinoma and adjacent tissues; (**B**) Relationship between the levels of PD-L1 and differentiation of hepatocellular carcinoma; (**C**) Repressive images of PD-L1 expression in hepatocellular carcinoma and adjacent tissues.

A total of 24 males and 4 females aged 40–78 years were included in the study, for a total 28 patients with HCC. Of the 28 total patients, six patients had poorly differentiated HCC, 19 patients had moderately differentiated HCC, and 3 patients had highly differentiated HCC according to pathological differentiation degrees. Moderately and highly differentiated patients were combined into one group due to a low sample size (n=3) in the study population. Immunohistochemistry was used to detect the expression of PD-L1 in liver cancer and adjacent tissues. Compared with adjacent tissues (1.29 ± 0.67), the expression of PD-L1 in liver cancer tissues was slightly higher (1.57 ± 0.90); however, no significant difference between the two groups was observed (t = 1.34, P > 0.05) (Figure 5A). The expression level of PD-L1 in the poorly differentiated HCC group was 1.75 ± 1.08 and 1.52 ± 0.87 in the medium and high differentiation group, with no significant differences observed between the two groups (t = 0.54, P > 0.05) (Figure 5B). Representative images of PD-L1 staining for each group are shown in Figure 5C. Ultimately, these data indicate that expression of PD-L1 is not dependent on the degree of HCC differentiation.

## DISCUSSION

Exosomes derived from cancer cells have a strong ability to alter the tumor microenvironment and have been implicated in driving epithelial mesenchymal transformation (EMT) in cancer cells [18, 19]. During the development of liver cancer, cancer cells secrete a large number of exosomes, which promote tumor initiation, angiogenesis, EMT, cellular matrix remodeling, and immune regulation, and thus support tumor progression and metastasis [20, 21]. Similar to liver cancer, exosomes secreted by HBV-infected hepatocytes play an important role in regulating the immune response to chronic HBV [22]. Kakizaki et al. [23] showed that exosomes secreted by HBV-infected cells strongly inhibited the immune response and clearance of virally infected cells in murine studies. In our study, the expression level of PD-L1 in circulating exosomes of patients with CHB and HCC was significantly higher than that of healthy controls. However, there were no significant differences observed in the expression level of PD-L1 in circulating exosomes between patients with CHB and HCC. This may be due to the variable range in expression levels of PD-L1 in circulating exosomes of patients with HCC. Mardomi et al. [24] recently found that PD-L1-overexpressing cardiomyocyte-like cells reduced CD4^+^ and CD8^+^T cell activation and infiltration *in vivo*. Differences between our results and those reported in the literature may be attributable to variations in the number of secreted exosomes and the levels of PD-L1 expressed due to inherent heterogeneity associated with HCC. In addition, other cells such as macrophages and dendritic cells can also release exosomes with PD-L1 except for cancer cells in HCC patients. Henceforth, it is necessary to expand the samples for the comparative study of patients with different stages of HCC.

A large number of immunosuppressive cell types have been identified in liver cancer tissues, such as bone marrow–like inhibitory cells and regulatory T cells, which secrete TGF-β and interleukin 10 (IL-10). These immunosuppressive cell types have been implicated in inhibiting the activity of immune cells, inducing CD8^+^T cell depletion and promoting tumor progression [25]. Zhou et al. [26] found that the expression of PD-1, T-cell immunoglobulin mucin-3 (TIM-3), and lymphocyte activation gene-3 (LAG-3) in lymphocytes in liver cancer tissues were significantly higher than that in lymphocytes residing in pericancerous tissues. Administration of anti-PD-L1, TIM-3, and LAG-3 antibodies could enhance proliferation and cytokine secretion by CD8^+^ and CD4^+^T cells. Wang et al. [27] reported that high levels of 14-3-3 ζ in exosomes from liver cancer cells depleted tumor-infiltrating T lymphocytes, reduced effector T cell activation and proliferation, reduced immune cell differentiation, and induced effector T cells to become Tregs. In this study, we observed that the expression level of CD107a in CD8^+^T cells in exosomes treated with anti-PD-L1 was significantly higher than that in the isotype control treated group, suggesting enhanced cytotoxic function of CD8^+^T cells after PD-L1 blockade. However, no significant effect on proinflammatory cytokine secretion of CD8^+^T cells was observed. Our results suggest that exosomal PD-L1 in HCC patients may interact with PD-1 expressed by CD8^+^T cells to inhibit CD8^+^T cell responses, ultimately leading to immune evasion and HCC disease progression. Therefore, blocking the binding of exosomal PD-L1 to PD-1 expressed by CD8^+^T cells in HBV-infected patients may enhance the production of proinflammatory cytokines by CD8^+^T cells, leading to immune clearance and reduced HBV-related liver damage.

The discovery and use of immune checkpoint blockade has significantly advanced immunotherapy treatment for cancer in recent years. It has been used in the treatment of many cancers, including liver cancer, and it has had some success in certain patient groups [28]. However, in most cancer types, only a handful of patients respond to and benefit from immune checkpoint blockade immunotherapy, likely due to either a lack of immunogenicity or the presence of heterogeneous immune microenvironments within tumors [29]. Based on the limited benefits associated with immunotherapy treatment, understanding why some patients respond and screening for associated biomarkers is vital to the advancement and future success of immunotherapy regimens. For example, the expression level of PD-L1 in tumor tissues correlates with patient response to immune checkpoint inhibitors [30, 31]. Our study found no significant difference in the expression of PD-L1 between liver cancer tissues and adjacent tissues, which is different from the results reported by Guo et al. [32]. These differences in observed outcomes might be attributable to differences in the patient cohorts and variations in detection methods. Alterations in adjacent tissues due to liver cirrhosis or chronic hepatitis infection can affect the expression of PD-L1. However, it is relatively difficult to obtain tumor tissue, and therefore relying on tumor tissues to detect PD-L1 expression is not conducive to dynamic detection [33], especially for HCC [34]. Li et al. [35] recently reported that exosomal PD-L1 expression correlated with the disease progression of non-small cell lung cancer. The level of circulating exosomal PD-L1 has been an early indicator of clinical benefit in melanoma patients treated with immune checkpoint inhibitors [36, 37]. Whether it has a similar prognostic role in patients with HCC warrants further exploration.

This study is the first to explore differences in the expression levels of PD-L1 in exosomes from patients with CHB and HCC, with a specific focus on their roles in immune clearance and immune escape. These results are preliminary, however, and the study has several limitations. First, our study had a small sample size and would have benefited from a larger patient population. Second, CD8^+^ T cell-mediated killing assay was not performed, only the expression of CD107a analyzed by FACS. However, CD107a expression cannot fully represent the cytotoxic activity of CD8^+^ T cells. Third, the detection method used to assess PD-L1 expression in exosomes might require further improvement.

In conclusion, the expression level of PD-L1 in the circulating exosomes of patients with HBV-related HCC was significantly increased. Antibody blockade of PD-L1 in exosomes might promote the cytotoxic function of CD8^+^T cells and might inhibit the occurrence and disease progression of HCC. On the contrary, blocking PD-L1 in exosomes *in vitro* might reduce the cytotoxic function of CD8^+^T cells in patients with chronic HBV and enhance the production of proinflammatory cytokines, which might contribute to immune clearance and reduced liver injury. This study might provide potential biomarkers for diagnosis and therapy of CHB and related HCC patients. However, this study is pilot, more experiment data are needed to validate our conclusion.
